# Age-related decreases in global metacognition are independent of local metacognition and task performance

**DOI:** 10.1016/j.cognition.2023.105389

**Published:** 2023-06

**Authors:** Andrew McWilliams, Hannah Bibby, Nikolaus Steinbeis, Anthony S. David, Stephen M. Fleming

**Affiliations:** a Wellcome Centre for Human Neuroimaging, Institute of Neurology, University College London, 12 Queen Square, London WC1N 3AR, UK; b Department of Experimental Psychology, University College London, 26 Bedford Way, London WC1H 0AP, UK; c Mental Health, Ethics and Law Research Group, Department of Psychological Medicine, Institute of Psychiatry, Psychology and Neuroscience, King's College London, Room 3.21, 16 De Crespigny Park, London SE5 8AF, UK; d Institute of Cognitive Neuroscience, University College London, Alexandra House, 17-19 Queen Square, London WC1N 3AZ, UK; e Institute of Mental Health, University College London, Wing A, 6th floor, Maple House, 149 Tottenham Court Road, London W1T 7NF, UK; f Max Planck Centre for Computational Psychiatry and Ageing Research, University College London, London, UK

**Keywords:** Local metacognition, Global metacognition, Metamemory, Self-efficacy, Domain-generality, Aging

## Abstract

Metacognition refers to a capacity to reflect on and control other cognitive processes, commonly quantified as the extent to which confidence tracks objective performance. There is conflicting evidence about how “local” metacognition (monitoring of individual judgments) and “global” metacognition (estimates of self-performance) change across the lifespan. Additionally, the degree to which metacognition generalises across cognitive domains may itself change with age due to increased experience with one's own abilities. Using a gamified suite of performance-controlled memory and visual perception tasks, we measured local and global metacognition in an age-stratified sample of 304 healthy volunteers (18–83 years; *N* = 50 in each of 6 age groups). We calculated both local and global metrics of metacognition and quantified how and whether domain-generality changes with age. First-order task performance was stable across the age range. People's global self-performance estimates and local metacognitive bias decreased with age, indicating overall lower confidence in performance. In contrast, local metacognitive efficiency was spared in older age and remained correlated across the two cognitive domains. A stability of local metacognition indicates distinct mechanisms contributing to local and global metacognition. Our study reveals how local and global metacognition change across the lifespan and provide a benchmark against which disease-related changes in metacognition can be compared.

## 1 Introduction

Metacognition refers to the capacity to reflect on and control other cognitive processes, or “think about thinking” (Dunlosky & Metcalfe, 2009; Flavell, 1979). The fidelity of metacognition is typically assessed by asking how subjective judgments - such as confidence - track objective performance (Fleming & Lau, 2014). Accurate metacognition is thought to optimise learning strategies, for instance, in education (Gascoine, Higgins, & Wall, 2017; Perry, Lundie, & Golder, 2019), by contributing to efficient cognitive offloading (Gilbert et al., 2020; Hu, Luo, & Fleming, 2019), or protecting against cognitive biases (Ackerman, Bernstein, & Kumar, 2020; Rollwage & Fleming, 2021). Metacognition is also thought to be compromised in neuropsychiatric illness (Bertrand, Landeira-Fernandez, & Mograbi, 2016; Hoven et al., 2019; Wells et al., 2010; White, Sadikot, & Djordjevic, 2016) and psychopathology (Rouault, Seow, Gillan, & Fleming, 2018; Seow, Rouault, Gillan, & Fleming, 2021), and has been related to fluctuating levels of insight (David, Bedford, Wiffen, & Gilleen, 2012).

Task-based measures of metacognition have enabled the precise measurement of relationships between confidence and objective performance in a range of cognitive domains (Ais, Zylberberg, Barttfeld, & Sigman, 2016). In such tasks, metacognitive “sensitivity” is defined as the trial-by-trial relationship between confidence and performance: good sensitivity is obtained when people report higher confidence when they are correct, and lower confidence when they are wrong. However, correlation-based metrics of metacognitive sensitivity (such as the gamma statistic) are susceptible to other confounds, such as variation in object-level performance (Fleming & Lau, 2014; Masson & Rotello, 2009). The development of the meta-*d’* model within a type 2 signal-detection theory framework has allowed metacognitive sensitivity to be quantified in a way that naturally controls for task performance, providing a metric known as metacognitive “efficiency”, or meta-*d’*/*d’* (Fleming, 2017; Galvin, Podd, Drga, & Whitmore, 2003; Maniscalco & Lau, 2012). Additionally, people undertaking tasks involving trial-by-trial ratings of confidence, such as during measurement of local metacognition, can be characterised by their tendency to give higher or lower confidence ratings in general, termed metacognitive bias. Biases in confidence again need to be accounted for when deriving local metacognitive efficiency parameters – a confound which can be approximately controlled for by applying type 2 signal-detection theory (Fleming, 2017; Maniscalco & Lau, 2012; Xue, Shekhar, & Rahnev, 2021).

We note that metacognitive sensitivity and bias are conceptually equivalent to the notions of resolution and calibration as often used in the judgment and decision-making literature (see Fleming & Lau, 2014, for a review of both approaches). These measures assume that confidence estimates are given on a probability scale, such that each scale point has objective meaning in terms of long-run success. Just as efforts have been made to correct measures of metacognitive sensitivity for differences in performance and bias, similar metrics (such as the adjusted normalized discrimination index, ANDI) have been developed in the probability judgment literature (Yaniv, Yates, & Smith, 1991). Finally, a recent metric grounded in information theory models metacognitive sensitivity as the mutual information between performance and metacognitive judgments (Dayan, 2022). Here, as our confidence scale was in arbitrary units, rather than probability increments, and we wished to capitalise on power afforded by the hierarchical meta-d’ model, we adopted the meta-d’ framework within which to estimate metacognitive efficiency and bias (Fleming, 2017).

A useful distinction is between “local” and “global” metacognition – where local metacognition refers to moment-to-moment appraisals of task performance, while global metacognition refers to long-run estimates of self-ability on specific tasks (Seow et al., 2021). Global metacognition shares common ground with notions of self-efficacy described in social psychology (Bandura, 1977), although the latter also encompasses aspects of controllability and mastery. Recent studies have shown that global and local metacognition rely on overlapping but distinct neural substrates (Rouault & Fleming, 2020), suggesting a hierarchy of metacognitive processes in the human brain (Seow et al., 2021). In neuropsychiatric illness these abilities may be impacted differently, with some evidence suggesting that local metacognition remains preserved despite changes in global metacognition in Alzheimer's disease (Gallo, Cramer, Wong, & Bennett, 2012). Similarly, we have recently documented intact local metacognition in patients with functional cognitive disorder, despite decreases in global confidence in memory performance (Bhome et al., 2022).

It remains uncertain how these distinct facets of metacognition vary with healthy aging, despite well-mapped trajectories for many other cognitive processes. Behavioural tasks involving trial-by-trial retrospective confidence judgments about performance provide initial evidence that local metacognition matures through adolescence (Fandakova et al., 2017; Moses-Payne, Habicht, Bowler, Steinbeis, & Hauser, 2021; Weil et al., 2013). In older adults, age-related atrophy of prefrontal and parietal regions (Resnick, Pham, Kraut, Zonderman, & Davatzikos, 2003) might be expected to bring about local metacognitive decline, due to an established role for frontoparietal networks in supporting local confidence estimates (see (Fleming & Dolan, 2012) for a review). On the other hand, it is also plausible that decades of experience of self and the world might actually bolster both global and local metacognitive abilities, creating more accurate self-appraisal and skilful use of strategies – such as in a prospective memory paradigm simulating daily life tasks (Aberle, Rendell, Rose, McDaniel, & Kliegel, 2010). Global self-appraisals of one's own abilities and function in different tasks and domains has also been studied with questionnaire measures of “self-efficacy”, including for memory ability specifically (Berry, West, & Dennehey, 1989; Zelinski & Gilewski, 2004). These measures of memory self-efficacy become increasingly negative with age (Cherry, Brigman, Reese-Melancon, Burton-Chase, & Holland, 2013; Hultsch, Hertzog, Dixon, & Davidson, 1988; Nyström, Sörman, Kormi-Nouri, & Rönnlund, 2019), and have been linked to beliefs about a declining ability to control memory in older age (Cherry et al., 2019; Lachman, 2006). However, how and whether such global self-appraisals are linked to alterations in local metacognitive processes, such as single-trial confidence formation, remains unknown.

Previous research into (local) metamemory in older age suggests it to be preserved despite task performance deteriorating. Confidence in encoding (prospective metacognition) has been studied with tasks involving prospective judgments of learning (JOLs) (Hertzog, Dunlosky, Powell-Moman, & Kidder, 2002; Robinson, Hertzog, & Dunlosky, 2006). Within these paradigms, older adults successfully predict their short-term memory span (Bertrand, Moulin, & Souchay, 2017), display improved accuracy in judgments of learning (JOLs) after a delay - as do younger adults - and appropriately update their confidence after studying new material (Connor, Dunlosky, & Hertzog, 1997; McGillivray & Castel, 2011; Rabinowitz, Ackerman, Craik, & Hinchley, 1982; Rhodes & Tauber, 2011). By recruiting participants across the adult lifespan (aged 18 to 81), Hertzog and colleagues showed that the resolution of JOLs in fact improved with aging (Hertzog, Sinclair, & Dunlosky, 2010). Similar sparing of metamemory capacity in older age has been documented for feeling-of-knowing (FOK) judgments (Butterfield, Nelson, & Peck, 1988; Marquié & Huet, 2000). Recent work capturing prospective confidence about learning has compared older and younger adults using signal-detection theory meta-d’ approaches, finding metacognitive efficiency to be preserved in older age (Zakrzewski, Sanders, & Berry, 2021). Monitoring of retrieval (retrospective metacognition) has also been studied using recognition memory tasks, finding both older and younger adults could judge how many items they had forgotten, although older adults had worse recognition performance (Halamish, McGillivray, & Castel, 2011). Work using retrospective confidence judgments – in other words, metacognition about retrieval – has also shown metacognitive capacity to be preserved in older age (Mitchell & Cusack, 2018).

Conversely, other research finds metamemory abilities deteriorate with age, both in tasks involving predicting learning and forgetting (Cauvin, Moulin, Souchay, Kliegel, & Schnitzspahn, 2019; Soderstrom, McCabe, & Rhodes, 2012) and in retrospective confidence judgments (Dodson, Bawa, & Krueger, 2007; Hertzog, Curley, & Dunlosky, 2021) with older adults more likely to be inappropriately overconfident. However, such effects on metamemory may be an epiphenomenon of age differences in memory (Dodson et al., 2007, Hertzog et al., 2021) – again emphasising the importance of controlling for first-order performance in studies of metacognitive ability.

There have been fewer studies of how perceptual metacognition changes with age. Signal detection modelling controlling for task performance and metacognitive bias has revealed deteriorating metacognitive efficiency in older age using retrospective confidence judgments, although within a relatively small sample of *N* = 53 participants (Palmer, David, & Fleming, 2014). Filippi, Ceccolini, Periche-Tomas, and Bright (2020) documented a trend towards impaired metacognitive efficiency in both childhood and older adults in a perceptual discrimination task, although first-order performance was also significantly lower in these groups, making interpretation difficult (Filippi et al., 2020). Another, recent, study compared younger (19–38 years old) and older (60–78 years old) adults (*N* = 30 in each group) on a laboratory psychophysical task that measured visual metacognitive sensitivity using contrast discrimination task together with a bias-free confidence-forced-choice procedure (De Gardelle & Mamassian, 2015; Klever, Mamassian, & Billino, 2022). They found reduced metacognitive sensitivity in older adults that was associated with lower composite executive function scores. However, there were also considerable individual differences in metacognitive sensitivity within each age group, and the older adults had overall lower contrast sensitivity meaning that the groups were not matched for task difficulty or first-order performance. In contrast, in a large web-based convenience sample there was no effect of age on local metacognitive efficiency in a staircased visual dot-discrimination task (Rouault, Seow, et al., 2018).

Whilst the lifespan trajectories of metacognition of memory and of perception remain to be fully characterised, a further question concerns the domain-generality of metacognition – the extent to which metacognitive ability in one domain predicts metacognitive ability in another domain (Rouault, McWilliams, Allen, & Fleming, 2018). Previous work has found evidence of domain-generality in local metacognition across sensory modalities (Faivre, Filevich, Solovey, Kühn, & Blanke, 2018) and between memory and perception (Mazancieux, Fleming, Souchay, & Moulin, 2020; McCurdy et al., 2013; Palmer et al., 2014). Other work has not found the same strong relationship at a behavioural level (Baird, Cieslak, Smallwood, Grafton, & Schooler, 2015; Baird, Smallwood, Gorgolewski, & Margulies, 2013; Morales, Lau, & Fleming, 2018), although neuroimaging and neuropsychological data suggest that both domain-specific and domain-general neural correlates of confidence may co-exist (Baird et al., 2013; Morales et al., 2018; Ye, Zou, Lau, Hu, & Kwok, 2018). However, although pioneering work in early childhood has suggested a transition to increasing domain-generality during development (Vo, Li, Kornell, Pouget, & Cantlon, 2014), the lifespan trajectory of domain-general and domain-specific components of metacognition remains unknown.

In the current pre-registered study, we set out to characterise a range of local and global metacognitive parameters across the adult lifespan by recruiting a general population sample of healthy volunteers stratified by age. Through use of staircase procedures to control performance levels on tasks, we aimed to identify either stability or change in both global and local metacognition in two cognitive domains, and dissociate such trajectories from an anticipated deterioration in first-order task performance in older age.

## 2 Methods

### 2.1 Design and pre-registration

We employed a cross-sectional design using novel measures of local and global metacognition in 2 cognitive domains (short-term memory memory and visual perception). The experimental design and planned analyses were pre-registered (see Open Science Framework; https://osf.io/6t7fn/) before data collection took place.

### 2.2 Participants

Volunteers were recruited from the academic crowdsourcing website, “Prolific” (https://www.prolific.co/; (Palan & Schitter, 2018, Peer, Brandimarte, Samat, & Acquisti, 2017). Potential participants were eligible if they were: 18 years of age or over, first language English, and had access to a Google Chrome or Safari browser on a desktop, laptop or tablet device. There were no exclusion criteria at recruitment, which was held open until 50 participants in each of 6 age groups (18–27 years, 28–37, 38–47, 48–57, 58–67, 68 years and over; see below for details) had completed all components of the experiment. The rate of pay was £7.50/h, with individual payments dependent on individual timings. If tasks were only partly completed or the session took longer than 3 h (predicted mean experiment time was 1 h), the participant was recompensed financially and their place re-allocated.

Sufficient power for reliable estimation of between-subject correlation coefficients can be obtained with a sample size of 150, based on a conservative anticipation of small effect sizes of ∼0.2 (Schönbrodt & Perugini, 2013). Individual group sizes of *N* > 20 have been shown in simulations as sufficient for robust hierarchical estimation of metacognitive parameters (Fleming, 2017). Our study sample is comfortably above these guidelines, with *N* = 50 in each of 6 age groups.

### 2.3 Measurement of local metacognition: development of “Metacogmission” and task structure

In order to ensure people remained motivated and engaged throughout our experiment, we developed a gamified, web-based environment to deliver performance-controlled perception and memory tasks, allowing us to collect trial-by-trial confidence judgments in two cognitive domains. We worked with a UK technology firm (*DamnFine* Ltd) and a mental health service user advisory group (The McPin Foundation) to develop “Metacogmission”: a suite of web-based behavioural tasks within a gamified environment, with accompanying back-story and stimulating visual content, whilst maintaining control over the psychophysical properties of the tasks (available using a Safari or Chrome browser at https://www.metacogmission.com/). Our iterative process of design and testing involved piloting over 400 people across the lifespan at two public engagement events - in an art gallery and at a music festival – where oral and written feedback on flow, graphics and intelligibility of instructions guided future design features. The mental health service user advisory group advised on levels of language complexity and accessibility features for marginalised groups. A fully functioning pilot version was administered in a clinical group with subjective cognitive impairment (Bhome et al., 2022).

**Fig. 1:**
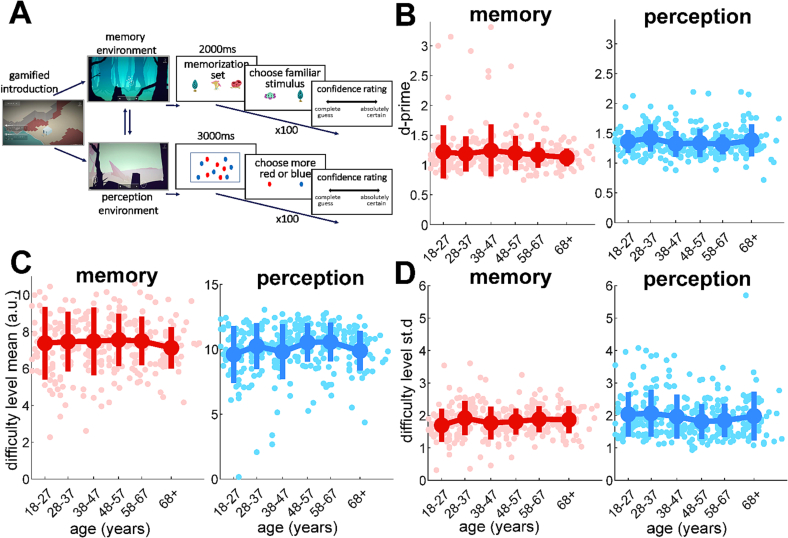
Task structure of Metacogmission and first-order task performance over the lifespan. A: Schematic of tasks measuring local metacognition for memory and perception. After a gamified introduction involving exploration of the planet environment, participants performed 100 two-alternative forced-choice trials in each domain. Memory trials involved presentation of a memorization set followed by a forced-choice short-term recognition memory judgment. Perceptual visual discrimination trials involved presentation of an array of red and blue dot-like shapes, followed by a choice about whether there had been more red or more blue shapes. Each trial was followed by a confidence rating using a slider. Task difficulty was adaptively staircased according to performance (see Methods) by altering the number of elements in the memorization set for memory or the difference in numbers of red and blue shapes for perception. B: Task performance (d’) plotted against age, with group means/standard errors for each age group. C and D: Levels achieved on the difficulty staircase for each participant reveal information about their performance capacity. Staircased difficulty levels are plotted against age, showing stable performance across age groups for both (C) mean difficulty level and (D) the standard deviation of difficulty level across trials. For the memory task, with larger set sizes creating greater difficulty, the plotted value is the mean number of items in the memorization set across the trials. In the perceptual task, larger differences between the numbers of blue and red shapes makes the task easier. Therefore, the perceptual difficulty value was generated by subtracting this difference from 15, so that larger difficulty values correspond to greater difficulty on both tasks. Individual points show subject-level data together with group means and standard errors. (For interpretation of the references to colour in this figure legend, the reader is referred to the web version of this article.)

Metacogmission has a gamified storyline, placing participants on an alien planet where they can explore and complete metacognitive memory and perceptual tasks to collect camp supplies (see Fig. 1A). Qualitative feedback received during task development at public engagement events indicated that the gamification and design elements made the experiment more attractive for many participants. This was especially the case for people who had not previously taken part in many cognitive science experiments, such as younger children and older adults. Short-term memory trials consist of a memorization set presented for 2 s, followed by a two-alternative forced-choice (2-AFC) requiring participants to select the familiar stimulus over a distractor presented beside it. Perceptual discrimination trials involved presentation of an array of multiple identical red and blue shapes for 3 s, followed by a choice of whether there were more red or blue stimuli.

(see https://github.com/metacoglab/McWilliamsBibbySteinbeisDavidFleming_AgeingMetacogmission2022 for the 5 sets of stimuli). For both memory and perception, retrospective confidence judgments were elicited after each trial using a horizontal visual sliding scale, the ends of which were labelled “complete guess” and “absolutely certain” (Fig. 1A). The pointer was placed initially in the centre and there were no visible divisions on the slider, generating confidence rating data on a quasi-continuous 201-point scale (coded in arbitrary units as −100 to +100).

Trials were presented in miniblocks of 20, with each miniblock having different thematic content. Each miniblock was preceded by additional optional practice trials. To aid engagement and motivation, at the end of each miniblock participants received feedback on their average accuracy and confidence in that miniblock. Participants were required to complete at least 5 miniblocks in each of the memory and perception domains to complete the task. If they submitted extra attempts at minblocks then these trials were also included in the analysis.

Importantly, in both cognitive domains, first-order task performance was controlled using a 2-down-1-up staircase procedure, which in the limit ensures first-order task performance converges to ∼71% correct (Levitt, 1971). The memorization set consisted initially of 2 stimuli, with the staircase increasing by 1 (after 2 consecutive correct trials) or decreasing by 1 (after 1 incorrect trial) the set size. The first perceptual discrimination trial showed a difference of 15 between the numbers of shapes of the 2 colours. The staircase then decreased (after 1 incorrect trial) or increased (after 2 consecutive correct trials) this difference by 1 to make the task harder or easier.

### 2.4 Measurement of global metacognition

Both before and after completing Metacogmission, global confidence ratings were obtained for each cognitive domain, through pre- and post-task self-reports of (expected) performance in the two domains compared to all other participants, using an 11-point Likert scale (0 “worse than everyone else” to 10 “better than everyone else”) (Rouault, Dayan, & Fleming, 2019). For the memory task, participants were told they would be asked to “remember and recognise some shapes or pictures”. For the perceptual task, they were informed they would need to decide “whether more red shapes or more blue shapes are shown on the screen”.

### 2.5 Analyses and data availability

Hierarchical Bayesian models were used to test pre-registered hypotheses about whether metacognitive efficiency changes with age and whether domain-generality changes or remains stable through life (Fleming, 2017). To analyse local metacognitive data generated from trial-by-trial confidence ratings, we fitted the meta-*d’* model within a signal detection theoretic framework (Maniscalco & Lau, 2012). Meta-*d’* is obtained by fitting a type 1 equal-variance Gaussian SDT model to the observer's empirical type 2 ROC. To obtain the empirical type 2 ROC, the conditional probabilities P(confidence = *y* | incorrect) and P(confidence = *y* | correct) are calculated for each confidence level; cumulating these conditional probabilities and plotting them against each other produces the type 2 ROC function (Clarke, Birdsall, & Tanner, 1959; Galvin et al., 2003). A type 2 ROC that bows sharply upwards indicates a high degree of sensitivity to correct/incorrect decisions (good metacognitive sensitivity). The area under the type 2 ROC (AUROC2) is itself a useful non-parametric measure of metacognitive sensitivity. However, AUROC2 is also expected to be affected by individual differences in type 1 performance (*d’* and criterion placement). By explicitly modelling the connection between performance and metacognition such potential confounds can be minimised. By fitting a type 1 SDT model to the observed type 2 ROC, we can determine the type 1 *d’* that best fits the observed confidence rating data. As this pseudo-*d’* is only determined by confidence data, and not the subject's type 1 performance, we label it meta-*d’*. Calculating the ratio meta-*d’*/*d’* then provides a metric of “metacognitive efficiency”, or “M-ratio”, which quantifies metacognitive sensitivity (meta-*d’*) relative to task performance (*d’*). Under an equal variance Gaussian signal detection theory model, meta-*d’* should be equal to *d’*, resulting in a theoretically optimal M-ratio of 1. For comprehensive descriptions of the meta-*d’* model and fitting process the reader is referred to (Maniscalco & Lau, 2012) and (Fleming, 2017).

Hierarchical Bayesian modelling within the HMeta-d toolbox was used for inference on these parameters at the group level, allowing direct group comparisons while avoiding reliance on noisy point estimates of single-subject parameters (Fleming, 2017). Certainty on these parameters (the group-level M-ratios) was determined by computing the 95% highest-density interval (HDI) from the posterior samples (Kruschke, 2010). Before entry into the hierarchical model, confidence ratings derived from the 201-point confidence scale were binned into 6 evenly spaced quantiles estimated for each individual within each cognitive domain. An extended version of the HMeta-d model was used to hierarchically estimate regression parameters relating metacognitive efficiency to covariates of interest such as age (Harrison et al., 2021). Performing multiple regression analyses within the HMeta-d model capitalises on the power of hierarchical estimation whilst avoiding problems encountered by post-hoc regressions on hierarchical model parameters, such as unwanted shrinkage to the group mean (Moutoussis, Hopkins, & Dolan, 2018).

To designate hits and false alarms for our 2AFC task, we arbitrarily selected one of the two responses (the response on the lefthand side of the screen) as “signal”. We calculated *d’* from hit and false alarm counts using the following formula:

$$d^{’}=z\left(\mathrm{HR}\right)–z\left(\mathrm{FAR}\right)$$

where z is the inverse of the standard cumulative normal distribution function, and HR and FAR refer to hit rate and false alarm rate respectively.

Although it might be expected that noise in the model fit could vary as a function of age, our analysis approach controls for the influence of this potential heterogeneity. When inferring group-level parameters, the model assigns less weight to single-subject contributions with a higher degree of uncertainty (Fleming, 2017). It is therefore possible that data from older adults become downweighted in the group estimate if their individual parameter estimates are noisier. Our analysis approach avoids such influences by fitting a separate group-level model to each of the 6 age groups. This precludes younger adult data from exerting a biasing influence on posterior estimates obtained for older adults.

All analysis code is publicly available, and links to the data (on the Open Science Framework) can be found at https://github.com/metacoglab/McWilliamsBibbySteinbeisDavidFleming_AgeingMetacogmission2022

## 3 Results

### 3.1 Participants and participant exclusions

305 participants completed all components of the study. Following our pre-registered criteria, a single participant (age group: 18–27 years) was excluded because they gave the same confidence rating for every trial in one domain, precluding estimation of metacognitive efficiency. 304 participants were therefore included in subsequent analysis and the demographics of the 6 age groups are shown in Table 1. Reported sex was female: *n* = 152; male: *n* = 150; non-binary: *n* = 0; ‘prefer not to say’: *n* = 2. A chi-squared comparison of male and female responses did not indicate that proportions differed between age groups (*p* = 0.12).

**Table 1:** Participant age group composition.

Group	Target age range (years)	n	Mean age (range; std)	Female⁎	Male⁎
1	18–27	49	23.1 (18–27; 2.89)	23 (48.0%)	25 (52.1%)
2	28–37	50	32.0 (28–37; 2.49)	17 (34.0%)	33 (66.0%)
3	38–47	50	42.0 (38–47; 2.78)	23 (47.0%)	26 (53.1%)
4	48–57	53	51.7 (48–57; 2.73)	35 (66.0%)	18 (34.0%)
5	58–67	51	60.7 (58–66; 2.49)	27 (52.9%)	24 (47.1%)
6	68 and over	51	72.2 (68–83; 3.90)	27 (52.9%)	24 (47.1%)
All (total)	18 and over	304	48.2 (18–83; 16.85)	152	150

⁎ percentage calculations exclude the 2 participants who did not state their sex

### 3.2 Preprocessing and data exclusions

Following our pre-registered criteria, 78/30,940 (0.0025%) memory trials and 35/26,300 (0.0013%) perceptual trials were excluded due to response times being longer than 30 s for either the first-order task judgment or metacognitive rating. The first 20 trials in each domain were removed for each participant prior to further analysis, to allow task difficulty staircases to stabilize.

### 3.3 First-order task performance and difficulty staircasing

To control task performance, continuous adaptive staircases made the perception and memory tasks more or less difficult in response to each subject's trial-by-trial performance. If the staircase was functioning well, we should expect to see similar levels of task performance across participants. We therefore plotted the task accuracy resulting from the difficulty staircase (defined as the signal-detection theory parameter *d’*) within each of the 6 age groups, as well as individual level data, showing that task accuracy was indeed stable across the lifespan (Fig. 1C). Linear regressions did not reveal a significant effect of age on *d’* for either memory (normalized beta mean − 0.08, s.e. 0.06, *p* = 0.19) or perception (normalized beta mean − 0.06, s.e. 0.06, *p* = 0.31), showing that staircasing was successful across the age range.

Since the staircase adjusts task difficulty to match each individual participant's performance, average task difficulty is itself of interest as a metric of first-order performance capacity. Therefore, we next asked whether the task difficulty levels which participants achieved on the staircase differed across the age range. Fig. 1C shows the mean staircased task difficulty within each of the 6 age groups (with higher values corresponding to greater difficulty). Notably, linear regression again revealed no effect of age either for memory (normalized beta mean − 0.03, s.e. 0.06, *p* = 0.65) or perception (normalized beta mean 0.07, s.e. 0.06, *p* = 0.21), indicating that both staircased task difficulty and observed performance levels were unrelated to age. Finally, Fig. 1D plots the standard deviation of trial-by-trial staircased difficulty levels for each subject – a performance variable that has been identified as a potential confound of metacognitive efficiency estimates (Rahnev & Fleming, 2019). Again, linear regressions show stability in staircase variability across the 6 age groups for both memory (normalized beta mean 0.09, s.e. 0.06, *p* = 0.13) and perception (normalized beta mean − 0.09, s.e. 0.06; *p* = 0.12).

### 3.4 Local metacognitive efficiency

**Fig. 2:**
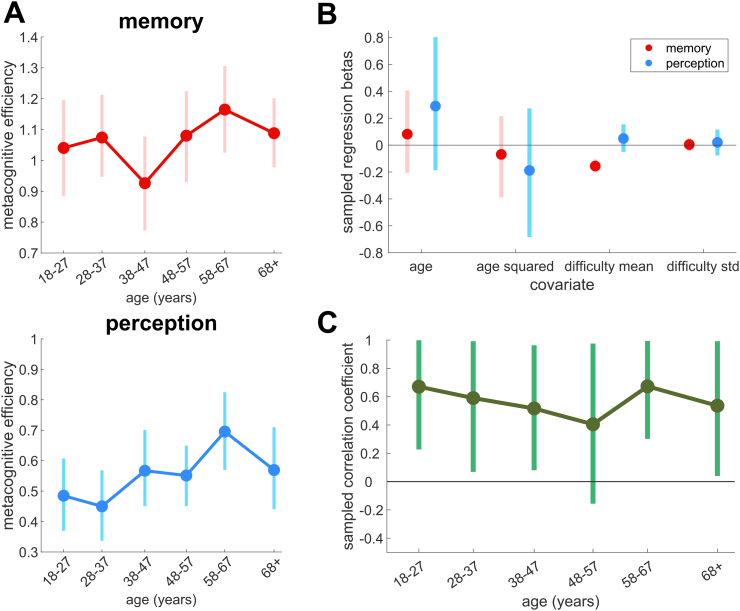
Relationships between age, local metacognitive efficiency and task performance. A: Estimates of group-level metacognitive efficiency (meta-*d’/d’*) are plotted for each of 6 age groups, for memory and for perception, showing the mean estimate and the 95% Highest Density Interval (HDI). B: Estimates of hierarchical multiple regression parameters predicting metacognitive efficiency for the entire cohort (*n* = 304), plotted as normalized betas, and showing means and 95% HDIs. Separate regressions were performed for memory (red) and for perception (blue). C: Domain-generality of metacognitive efficiency across the lifespan. Hierarchical estimates within each age group of the covariance between memory and perception metacognitive efficiency, showing mean parameter estimates and the HDIs. In all but one age group (48–57 years), the HDI is positive and does not overlap zero, indicating a significant correlation between domains. (For interpretation of the references to colour in this figure legend, the reader is referred to the web version of this article.)

We next computed local metacognitive efficiency (meta-*d’*/*d’*) from trial-by-trial confidence ratings. Within each age group, for memory and for perception in turn, group-level estimates of local metacognitive efficiency were derived using the HMeta-d toolbox (Fleming, 2017) (Fig. 2A). Certainty on these parameters (the group-level M-ratios) was determined by computing the 95% HDIs from the posterior samples of estimates of the group mean (Kruschke, 2010). Notably, the overlapping HDIs indicate only limited change in local metacognitive efficiency from age group to age group, both for memory and for perception (Table 2).

**Table 2:** Metacognitive efficiency parameter estimates.

Age group	Group metacognitive efficiency (meta-*d’*/*d’*)		Correlation coefficient (mean, 95% HDI⁎)
	Memory (mean, 95% HDI⁎)	perception (mean, 95% HDI⁎)	
1	1.04, [0.88, 1.20]	0.48, [0.37, 0.61]	0.67, [0.23, 1.00]
2	1.07, [0.95, 1.21]	0.45, [0.34, 0.57]	0.59, [0.07, 0.99]
3	0.93, [0.77, 1.08]	0.57, [0.45, 0.70]	0.52, [0.08, 0.96]
4	1.08, [0.93, 1.22]	0.55, [0.45, 0.65]	0.40, [−0.16, 0.98]
5	1.16, [1.03, 1.31]	0.70, [0.57, 0.83]	0.67, [0.30, 0.99]
6	1.09, [0.98, 1.20]	0.57, [0.44, 0.71]	0.54, [0.04, 0.99]
All ages	1.07, [1.01, 1.12]	0.55, [0.49, 0.60]	0.60, [0.38, 0.79]

⁎ 95% highest-density interval of posterior samples

We next asked whether age statistically predicted metacognitive efficiency by entering age as a predictor in a hierarchical multiple regression model of meta-d’/d’ across the entire subject pool (n = 304) (Harrison et al., 2021). We performed separate regressions for memory and perception, with linear and quadratic age terms, as well as covariates for features of first-order performance (mean and standard deviation of staircased difficulty). Mean estimates of normalized betas are plotted with their 95% HDIs in Fig. 2B. We found no effect of age on metacognitive efficiency, either for memory (age beta mean: 0.08; HDI: [−0.20, 0.41]; age-squared beta mean: -0.07; HDI: [−0.39, 0.21]) or perception (age beta mean: 0.29; HDI: [−0.18, 0.80]; age-squared beta mean: -0.19; HDI [−0.68, 0.27]). We note that while these effects of age were not statistically significant, their sign was in general positive (Fig. 2A and B), and therefore provides robust evidence against a deterioriation of local metacognition with age. Turning to associations with task difficulty, memory task difficulty showed a negative relationship with metacognitive efficiency (beta mean: -0.16; HDI: [−0.20, −0.11]), though this effect was not found for perception (beta mean: 0.05; HDI: [−0.05, 0.15]). No effects were found of variability in task performance (calculated as the standard deviation of the staircased difficulty achieved within each subject's trials) on metacognitive efficiency, for either memory (beta mean: 0.004; HDI: [−0.04,-0.05]) or perception (beta mean: 0.02; HDI: [−0.08,0.11]) – suggesting such variability was not confounding our estimates of metacognitive parameters (Rahnev & Fleming, 2019).

We next explored the extent to which metacognitive efficiency was domain-general across the age groups. A common approach to estimating domain-generality is to calculate the extent to which metacognitive efficiency (i.e., metacognitive sensitivity corrected for influences of task performance) is correlated across domains. We undertook Bayesian hierarchical estimations of the group cross-task covariance between individual-level metacognitive efficiencies for the two cognitive domains (Fleming, 2017). Parameter estimates of the cross-task correlation that are greater than zero provide evidence of a positive covariance between the domains. An analysis treating all participants as a single group (*n* = 304) generated a mean estimate for this correlation coefficient of 0.60, with the 95% HDI above zero (HDI: [0.38, 0.79]), in line with other recent estimates of this effect (Mazancieux, Fleming, Souchay, & Moulin, 2020). Next, we performed this analysis for each of the 6 age groups individually, to ask how this cross-task correlation (domain-generality) itself may alter with age. Mean estimates for each age group are plotted with their 95% HDIs in Fig. 2C, with the values shown in Table 2. All 6 mean estimates were >0.4, with a significant positive correlation (indicative of domain-generality) in 5/6 age groups (the exception was the 48–57-year-olds, although we note the shape of the posterior distribution was similar to the other groups and positive in 89% of samples, indicative of a false negative). This positive correlation also remained broadly stable across the adult lifespan, as evidenced by the highly overlapping 95% HDIs on the correlation parameter from age group to age group.

### 3.5 Local metacognitive bias

**Fig. 3:**
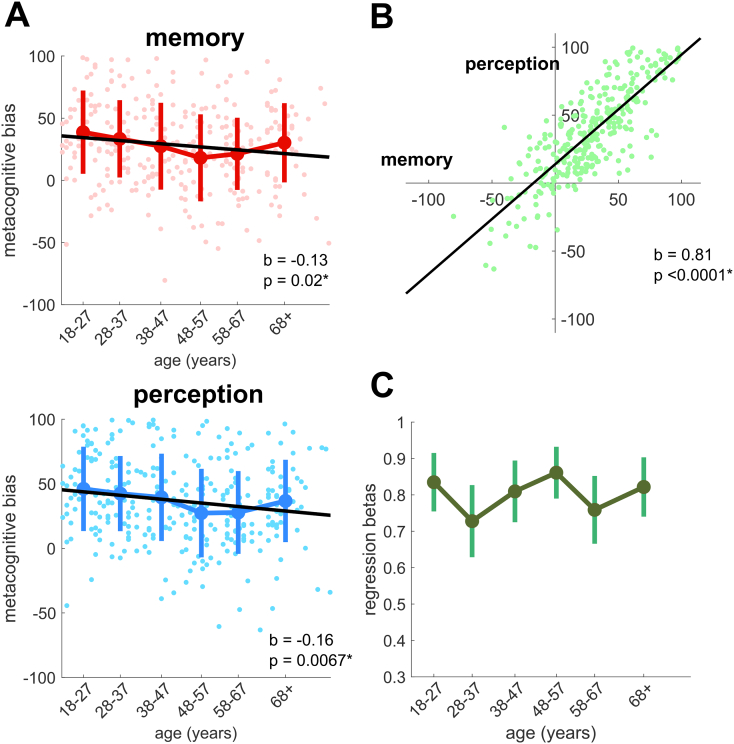
Relationships between age and local metacognitive bias. Confidence on a sliding scale was coded to range between −100 to +100. A: Mean confidence for individual participants is plotted for each cognitive domain, overlaid with group mean confidence for each of the 6 age groups, with error bars indicating standard error of the mean. B: Domain-generality of metacognitive bias over the whole cohort (*n* = 304), quantified by plotting the correlation between individual level mean confidence on the memory and perceptual tasks. The solid line shows the best-fitting linear regression. C: Domain-generality of metacognitive bias within each of the 6 age groups was estimated by calculating the correlation between memory and perception confidence within each age group separately. Plotted are the normalized betas from linear regression models estimating the relationship between perceptual and memory confidence within each age group, with error bars indicating standard error of the mean.

We next calculated another key feature of local metacognition known as “metacognitive bias” – the mean confidence rating given by individual participants across all trials. Note that although the visual sliding scale had ends labelled “complete guess” and “absolutely certain”, participants' ratings were collected on an arbitrary 201-point scale, with −100 corresponding to “complete guess” and + 100 to “absolutely certain”. Taking each cognitive domain in turn, the group mean values and standard errors within each of the 6 age groups are plotted in Fig. 3A, together with individual level data. We performed a linear regression on age within each cognitive domain, finding a significant negative effect of age on metacognitive bias for both memory (normalized beta mean: −0.13, s.e.: 0.06, *p* = 0.02) and perception (normalized beta mean: −0.16, s.e.: 0.06, *p* = 0.0067). This result indicates that while first-order task performance and local metacognitive efficiency are both stable across the age range, people's average confidence tends to decrease into older age, in both of our two task domains. We also analysed the variability (standard deviation) of participants' confidence ratings as a function of their age (supplementary figure), showing no clear relationship. This indicates older adults were just as willing as younger adults to use the full range of the confidence scale.

In order to explore the extent to which metacognitive bias is domain-general, we analysed the relationship between mean confidence levels in perception and memory across participants. A scatter plot of this relationship revealed a strong and significant positive correlation (normalized beta 0.81, s.e. 0.03, *p* < 0.0001; Fig. 3B), in keeping with previous findings (Ais et al., 2016; Mazancieux, Dinze, Souchay and Moulin, 2020). Performing linear regressions within each of the 6 age groups individually showed that a domain-generality in metacognitive bias was maintained across the lifespan (Fig. 3C).

### 3.6 Global metacognition

**Fig. 4:**
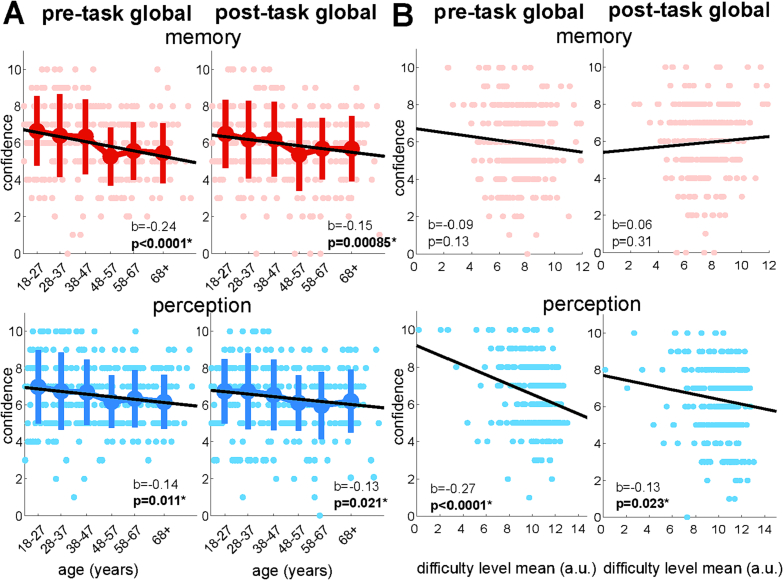
Relationships between age and global metacognition. Before and after both the memory and perception tasks, participants were asked to give an overall rating of how well they thought their task performance would compare to other participants, using an 11-point Likert scale (0−10). A: Relationships with age. For memory and perception, pre- and post-task global self-performance estimates (SPEs) are plotted for each of the 6 age groups, with per-group means and standard errors. Additionally, individual-level data are shown, with linear regressions on age. B: Relationships with task performance. Again, for memory and perception, pre- and post-task global SPEs are plotted, regressed against the difficulty achieved on the staircasing procedure (higher values correspond to greater difficulty in both tasks).

Finally, we analysed our measures of pre- and post-task global self-performance estimates (SPEs) for memory and perception. These ratings can be considered as measures of global metacognitive bias, since they elicit a metric of overall confidence in long-run performance, in the same manner as our measures of local metacognitive bias. We note here we are unable to relate these global estimates to objective fluctuations in local performance, unlike alternative task-based approaches to global metacognition (Lee, De Gardelle, & Mamassian, 2021; Rouault et al., 2019). We plotted pre- and post-task SPEs within each of the 6 age groups for each task separately (Fig. 4A). We found negative effects of increasing age on memory SPEs both pre-task (normalized beta mean: −0.24, s.e.: 0.06, *p* < 0.0001) and post-task (normalized beta mean: −0.15, s.e.: 0.06, *p* = 0.0085), and a similar negative effect of age on perception SPEs both pre-task (normalized beta mean: −0.14, s.e.: 0.06, *p* = 0.011) and post-task (normalized beta mean: −0.013, s.e.: 0.06, *p* = 0.021). This result is again consistent with an effect of age on (local and global) metacognitive bias, with older adults being less confident in their performance both before, during and after a task, despite their first-order performance and local metacognitive efficiency remaining unchanged.

We next examined the degree to which global SPEs changed after completing the tasks at a within-participant level. Both for memory and for perception in turn, pre- and post-task global SPEs did not on average differ within each participant (paired *t*-tests: memory, *p* = 0.91; perception, *p* = 0.20). In addition, when we regressed individual changes in global SPEs from pre- to post-task on age, no relationship was found, either for memory (normalized beta mean: 0.10, s.e.: 0.06, *p* = 0.083) or for perception (normalized beta mean: 0.0086, s.e.: 0.06, *p* = 0.88).

We also explored relationships between indicators of objective task performance (quantified by staircased difficulty level) and global metacognition (Fig. 4B). For the memory task, linear regressions on the mean staircase difficulty level did not show a significant relationship, either with pre-task (normalized beta mean: −0.09, s.e.: 0.06, *p* = 0.13) or post-task SPEs (normalized beta mean: 0.06, s.e.: 0.06, *p* = 0.31). However, in the perception task, people who had better task performance had lower global SPEs, with pre-task SPEs showing a negative linear relationship with task difficulty (normalized beta mean: −0.27, s.e.: 0.06, *p* < 0.0001). The same was true for post-task SPEs, although to a lesser degree (normalized beta mean: −0.13, s.e.: 0.06, *p* = 0.023).

As our visualization of global memory SPEs showed trends (in Fig. 4) towards a negative association with staircased difficulty pre-task, but a positive association with staircase difficulty post-task, we performed an additional exploratory analysis to formally test for this interaction. Specifically, we asked whether the degree to which individuals updated their global SPE from pre- to post-task was related to memory staircase difficulty, finding a positive linear relationship (normalized beta mean: 0.17, s.e.: 0.06, *p* = 0.0024). This could suggest that people were more likely to boost their post-task SPE if they were able to achieve a higher level of task performance (greater task difficulty) during the experiment. A similar relationship was also found when we looked at participants' updating of global SPEs on the perceptual task (normalized beta mean: 0.14, s.e.: 0.06, *p* = 0.018).

Finally, we asked whether participants' global SPEs in memory and perception were domain-general. When taking an aggregate measure of global metacognition in each domain (mean of pre- and post-task confidence rating), we found a strong positive linear relationship between global estimates in the perception and memory tasks (normalized beta mean: 0.73, s.e.: 0.04, *p* < 0.0001). Additionally, the extent to which participants' tended to update their global SPEs from pre- to post-task was correlated between the two domains (normalized beta mean: 0.36, s.e.: 0.05, p < 0.0001).

## 4 Discussion

Previous studies charting changes in metacognition through adulthood have drawn conflicting conclusions about relationships with age. Our large-scale web-based study used an age-stratified sample (in groups of *N* = 50 equally distributed from 18 to 83 years) to explore how local and global aspects of metacognition for memory and perception relate to age. We obtained several key findings. First, we found that local metacognitive efficiency (the accuracy with which trial-by-trial confidence ratings track performance) was stable across the lifespan, consistent with other work showing that local metacognitive capacity is relatively preserved into older age (Bertrand et al., 2017; Butterfield et al., 1988; Connor et al., 1997; Halamish et al., 2011; Hertzog et al., 2002; Hertzog et al., 2010; Hertzog & Dunlosky, 2011; McGillivray & Castel, 2011; Rhodes & Tauber, 2011; Robinson et al., 2006; Rouault, Seow, et al., 2018; Souchay, Moulin, Clarys, Taconnat, & Isingrini, 2007). Notably, however, we found that metacognitive bias (participants' average confidence judgments about their performance, irrespective of accuracy) became more negative with age, as did self-performance estimates about one's ability to perform entire tasks (an aspect of global metacognition) These age-related decreases in confidence were not accompanied by objective changes in performance, which remained stable across the lifespan. Finally, both local and global aspects of metacognition tended to be correlated across domains, and this correlation was stable into older age.

Given the established relationship between prefrontal function and metacognition, and frontal lobe changes in aging (Fleming, Ryu, Golfinos, & Blackmon, 2014; West, 1996), it might be expected that metacognition would also decay in older age. However, our finding that local metacognitive efficiency for memory or perception is stable across the lifespan is consistent with other prior work (Connor et al., 1997; Halamish et al., 2011; Hertzog et al., 2021; Hertzog & Hultsch, 2000; Zakrzewski et al., 2021). Our procedures used trial-by-trial staircasing of task difficulty to control subjects' task performance within a narrow range, allowing effective isolation of individual differences in metacognitive efficiency (Fleming, Weil, Nagy, Dolan, & Rees, 2010; Levitt, 1971). By ensuring participants in all 6 age groups were performing with the same level of accuracy on the memory and perception tasks (as shown by stability in *d’*), we were able to derive estimates of metacognitive sensitivity which were unlikely to be confounded by the information available for confidence judgments (as quantified by *d’*). In addition, use of the hierarchical meta-*d’* model allowed us to obtain a performance-corrected estimate of metacognitive efficiency, meta-*d’/d’*, for each age group. Estimating this group-level parameter within a hierarchical model that combined data from each group's *N* = 50 subjects afforded particularly precise estimates on age-related changes in metacognitive efficiency, as such a model fit is able to capitalise on at least *N* = 10,000 confidence ratings per age group (N = 50 × 200 trials per subject (Fleming, 2017)).

We note that there are conflicting findings on the effects of age on local metacognitive efficiency. Early studies from our lab found that the meta-*d’/d’* ratio in a near-threshold psychophysics task (two-interval Gabor contrast discrimination) increased during adolescence (Weil et al., 2013) but then declined into older age (Palmer et al., 2014). Another recent study using a bias-free confidence-forced choice task to assess metacognition of visual perception found metacognitive sensitivity to be diminished in older age, with this effect associated with composite executive function scores (Klever et al., 2022). One possibility is that these laboratory samples are more representative of the older population given that online study samples are likely to select for more technology literate older adults (see Limitations below). Alternatively, it is possible that there are first-order performance limitations in inherently challenging laboratory psychophysical tasks may limit the extent to which older adults can reveal their intact metacognitive capacity. Consistent with this perspective, Palmer et al. found that reductions in metacognitive efficiency with age – calculated as the meta-*d’/d’* ratio – were driven more by increases in *d’* than by decreases in meta-*d’*, perhaps as a result of the adaptive staircase procedure creating relatively easier tasks for the older adults (Palmer et al., 2014). Conversely, in Klever et al.'s study, the older age group showed lower contrast sensitivity, implying they were faced with a more difficult perceptual task than the younger adults.

There is longstanding debate about the extent to which laboratory delivery of studies fails to capture something about real-world (meta-)cognition, and our gamified task environment, developed in conjunction with a mental health service user advisory group, represents a first step in bridging this divide (Shamay-Tsoory & Mendelsohn, 2019). Preservation of metacognitive efficiency into older age is instead consistent with the idea that older people develop knowledge about their own cognitive capacities and strategies, leading to preserved metacognition (Hertzog et al., 2010; Hertzog et al., 2021; Hertzog & Dunlosky, 2011). Self-knowledge of one's behavioural and cognitive limitations, and of effective strategies for approaching cognitive tasks may in turn go some way to account for the preservation of first-order performance seen in older adults undertaking tasks in naturalistic, rather than laboratory, settings (Aberle et al., 2010; Rendell & Craik, 2000).

Preservation of first-order performance on our memory task may not be too not surprising, given evidence that performance on short-term memory tasks (as opposed to working memory tasks) has been shown to be spared with aging, especially in the absence of competing demands or interference (Bopp & Verhaeghen, 2009; Hoefeijzers, González Hernández, Magnolia Rios, & Parra, 2017; Verhaeghen, 2011). A central reason for adopting a visual short-term memory task is that it allowed us to manipulate memorization set size from trial to trial, thus clamping first-order performance and ensuring comparability with the measurement of metacognition in the perceptual domain. It is more challenging to staircase longer-term memory tasks in which the memorisation phase occurs infrequently.

It is possible that the lack of age effect on memory metacognitive efficiency in the current study was related to the idiosyncratic nature of our task, which likely involved short- to medium-term and recognition memory processes (Bertrand et al., 2017). In particular, smaller memorization sets might be handled within short-term memory alone (which is thought to be preserved into older age). There could also be within-participant effects, where different types of memory – and even use of distinct strategies – become more important at different set sizes. Notably, other studies have reported that older adults show changes in metacognition relative to younger adults on episodic but not semantic memory tasks – although such alterations may be partially attributed to first-order factors such as memory for target-related information (Souchay et al., 2007). More broadly, our study highlights a need to understand how metacognition of other forms of memory, such as episodic recall, may alter in older age (Mazancieux, Dinze, Souchay, & Moulin, 2020; Souchay, Guillery-Girard, Pauly-Takacs, Wojcik, & Eustache, 2013).

Our study replicated previous findings that metacognition exhibits a significant domain-general component, since both metacognitive efficiency and bias for memory and perception tended to covary across tasks (Ais et al., 2016; Morales et al., 2018; Rouault, McWilliams, et al., 2018). We extend this line of research to document the change in cross-domain covariance with age, finding that domain-generality was already established in our youngest age group (18–27 years old) and remained stable into older age. Notably, previous work on the early development of metacognition has suggested it to be more domain-specific in childhood: only limited association between metacognitive abilities measured across tasks has been shown in 5- to 8-year-olds undertaking visual numerical and emotional discrimination tasks, or in 6- to 7-year-olds undertaking reading and emotional tasks (Taouki, Lallier, & Soto, 2021; Vo et al., 2014). Given that metacognition continues to develop through childhood and adolescence (Fandakova et al., 2017; Weil et al., 2013), it remains to be determined if and when a transition from domain-specific to domain-general metacognitive capacities occurs during this period.

One concern when interpreting domain-general correlations in local and global metacognitive bias (average confidence) is that such effects may be driven by individual differences in confidence scale usage, such as idiosyncrasies in the selection of scale anchor points (Foda, Barger, Navajas, & Bahrami, 2017). In the current study we are unable to eliminate the influences of such effects on our results. However, we note that other studies have shown systematic relationships between explicit confidence ratings and other behavioural indices of confidence-related behavior, such as offloading tendency (Boldt & Gilbert, 2019; Hu et al., 2019) and information seeking (Schulz, Rollwage, Dolan, & Fleming, 2020). Including such measures in future studies of local and global metacognitive bias in aging would be of interest. Finally, such scale effects are unlikely to affect the assessment of domain-generality in metacognitive efficiency, which quantifies the across-trial coupling between confidence and performance, rather than an average rating over many trials. However here too there may be a number of factors affecting domain-generality in metacognitive sensitivity across tasks, such as the fidelity of post-decisional accumulation (Desender, Vermeylen, & Verguts, 2022) or unmodeled contributions to local metacognitive bias (Xue et al., 2021).

In contrast to this stability of metacognitive efficiency across the age range, a key finding was that both global metacognitive bias (pre- and post-task ratings of performance) and local metacognitive bias (average confidence during the task) became more negative with age, despite objective measures of task performance remaining stable. In other words, as we get older, we tend to have lower metacognitive expectations of our performance, in the near future (pre-task global ratings), the present (local metacognitive bias) and the recent past (post-task global ratings) – even if this is not objectively the case, relative to others. Such findings are consistent with other work showing decreases in memory self-efficacy into older adulthood (Cherry et al., 2013; Cherry et al., 2019; Hultsch et al., 1988; Nyström et al., 2019). Rather than assuming older adults are becoming inappropriately underconfident, an alternative explanation is that younger people are in fact inappropriately overconfident and that some aspects of metacognition may then re-calibrate with age. Interestingly, other work has shown that optimistic biases when updating beliefs about the likelihood of undesirable events may in fact increase with age (Chowdhury, Sharot, Wolfe, Düzel, & Dolan, 2014; Sharot, 2011). It remains to be seen how such an age-related positivity bias relates to the increasing negativity observed here when assessing aspects of cognitive self-performance. Our task design precludes analysis of whether the effect is driven by optimism in youth or pessimism in maturity, as global performance estimates were elicited relative to others, rather than relative to objective performance. Novel task-based methods for assessing global metacognitive bias and efficiency may allow this question to be answered in future studies (Hoven et al., 2022; Rouault et al., 2019).

The reasons for finding decreases vs. increases in confidence in older age remain to be determined – but one key factor could be whether objective performance is adequately accounted for. Indeed, recent work has suggested that overconfidence effects in memory tasks may be an epiphenomenon of age-related changes in memory, rather than metamemory (Hertzog et al., 2021), with poorer recollection of contextual details leading directly to an over-generous misappraisal of memory performance (Dodson et al., 2007; Hertzog & Dunlosky, 2011; Soderstrom et al., 2012). Here, in a large age-stratified sample using tasks that were matched for performance using staircase procedures, we found global and local confidence *decreased* with age, despite both performance and local metacognitive efficiency remaining stable.

The increased negativity of self-performance estimates we observed could be the result of age-related changes in responses to the social environment or societal views about the aging brain, such as those promoted purposefully or inadvertently by public health campaigns (Commissaris, Verhey Jr, Ponds, Jolles, & Kok, 1994; Heger et al., 2020; Hertzog, Small, McFall, & Dixon, 2019; Kinzer & Suhr, 2016; Maxfield & Greenberg, 2021). Older adults have been shown to score more highly on self-report assessments of social desirability (Soubelet & Salthouse, 2011), meaning older adults might wish to show modesty or humility in their confidence ratings. In turn, with increasing awareness of trajectories of typical aging and the incidence of neurodegenerative disease, older adults may both anticipate poor performance as being inevitable and recall it as having been so. Future research on these effects could study metacognition in people with life partners who have dementia, as exposure to such an illness might entail over-expectation of cognitive decline. Such concern about one's own cognition might eventually reach problematic levels, manifesting as health anxiety, subjective cognitive decline or functional cognitive disorder (Ball, Swirski, Newson, Coulthard, & Pennington, 2021; Divers et al., 2021; Hao et al., 2017). In keeping with this hypothesis, we recently measured metacognitive efficiency in functional cognitive disorder using a version of the current task, showing that participants had substantially lower confidence on global rating scales despite local metacognition remaining intact (Bhome et al., 2022).

### 4.1 Limitations

A cross-sectional design cannot study longitudinal change, including whether events in societal development (period effects such as in the education system or the arrival of the internet) have affected age cohorts differently. Despite the recent popularity and many benefits of web-based participation, it is important to be mindful of potential systematic biases in recruitment and participation which were not fully mitigated by our exclusion criteria.

One important consideration concerns whether the composition of the groups varies with age. Specifically, older adults who volunteer to take part in web-based psychological studies are likely to be the more technology literate members of their age bracket, compared to younger groups (Czaja et al., 2006). Our task was designed to be usable by a broad population cohort in both clinical and basic science studies of metacognition (Bhome et al., 2022), and was developed iteratively for acceptability and usability together with a mental health service user group. However, we cannot rule out that, in the current study, recruitment of our older adult sample via Prolific may have led to more high-functioning individuals in the older age brackets than in the broader population, and an underestimation of age differences in local metacognitive efficiency. Conversely, our web-based delivery of a gamified experiment via an academic crowdsourcing site allowed the participation of people who might not have been reached by standard recruitment processes or been able or interested to attend laboratory testing. Because Prolific aims to offer diversity by ethnicity, socio-economic background and employment status, our findings may also be more generalisable across the population than traditional psychological research, which has often struggled to recruit beyond a pool of university student participants.

## 5 Conclusions

Whether or not metacognition alters as we age has remained controversial. Here, in a large-scale online study we show that both global and local confidence decline with age, even though both local metacognitive efficiency and task performance remained stable. This finding generalized across both memory and perceptual tasks. A key influence of age on metacognition is that people think they are performing worse, even if this is not the case. In contrast to these systematic effects of age on overall confidence, local metacognitive efficiency – the ability to distinguish correct from incorrect trials – remained stable over the lifespan. While the current study sought to answer specific questions about metacognition and aging in the general population, these age-stratified data also provide a benchmark for metacognitive function in health, mental illness and neurodegeneration. Our findings indicate that alterations in metacognition, rather than primary abilities, may be one source of increasing subjective concern about cognitive ability with aging.

## CRediT authorship contribution statement

**Andrew McWilliams:** Conceptualization, Methodology, Investigation, Data curation, Software, Formal analysis, Visualization, Writing - original draft, Writing - review & editing. **Hannah Bibby:** Methodology, Investigation, Data curation, Writing – review & editing. **Nikolaus Steinbeis:** Methodology, Supervision, Resources, Writing - review & editing. **Anthony S. David:** Methodology, Supervision, Resources, Funding acquisition, Writing - review & editing. **Stephen M. Fleming:** Conceptualization, Methodology, Software, Formal analysis, Supervision, Resources, Funding acquisition, Writing - review & editing.

## Declaration of Competing Interest

None.

## Data Availability

Links to the data (on the Open Science Framework) can be found at https://github.com/metacoglab/McWilliamsBibbySteinbeisDavidFleming_AgeingMetacogmission2022

## Appendix A Supplementary data

Supplementary material. Supplementary figure: Standard deviation of raw confidence ratings by age. Image 1
