# Supplementary material for: Age-related decreases in global metacognition are independent of local metacognition and task performance
Source: Cognition. 2023 Jun;235:105389. doi: 10.1016/j.cognition.2023.105389 (PMC10632679; doi:10.1016/j.cognition.2023.105389)
Supplement: Supplementary file 1 — Supplementary material. Supplementary figure: Standard deviation of raw confidence ratings by age. [file mmc1.pdf]

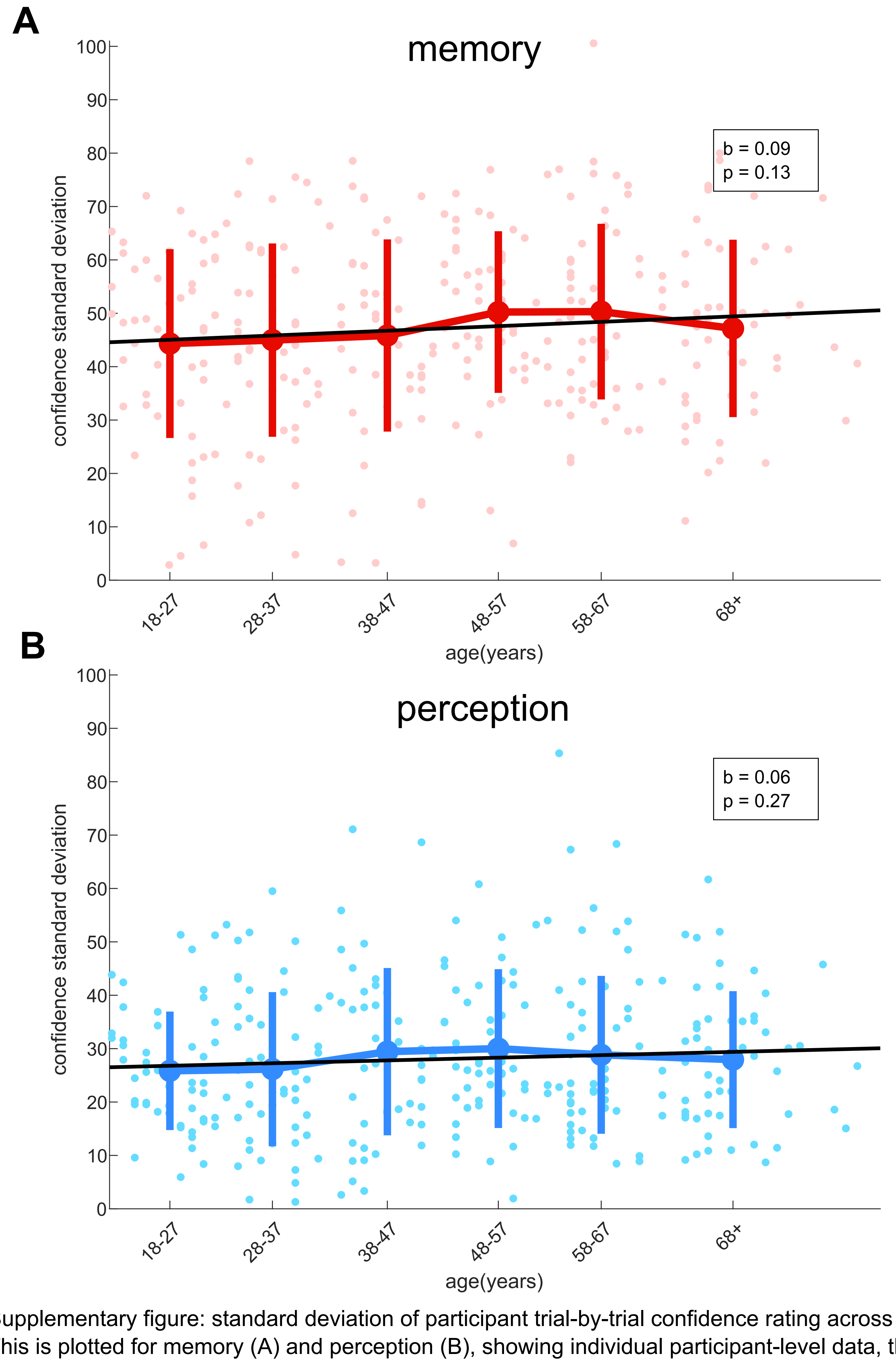

Supplementary figure: standard deviation of participant trial-by-trial confidence rating across all trials. This is plotted for memory (A) and perception (B), showing individual participant-level data, the mean value within each of the 6 age groups, with error bars showing standard errors of the means. A linear regression line is also fitted, with the beta values and p-values of this regression indicated.
